# O.1.2-9 Sex-related differences in free-living estimates of physical activity energy expenditure between older men and women

**DOI:** 10.1093/eurpub/ckad133.096

**Published:** 2023-09-11

**Authors:** Laura Karavirta, Timo Rantalainen, Kate Westgate, Timo Aittokoski, Joona Neuvonen, Jukka A Lipponen, Katri Turunen, Riku Nikander, Erja Portegijs, Taina Rantanen, Soren Brage

**Affiliations:** University of Jyväskylä, Finland; University of Jyväskylä, Finland; University of Cambridge, UK; University of Jyväskylä, Finland; University of Jyväskylä, Finland; University of Eastern Finland, Finland; University of Jyväskylä, Finland; Seinäjoki University of Applied Sciences, Finland; University of Jyväskylä, Finland; Wellbeing region of Central Finland, Finland; University of Jyväskylä, Finland; University of Groningen, The Netherlands; laura.i.karavirta@jyu.fi; University of Jyväskylä, Finland; University of Cambridge, UK

## Abstract

**Purpose:**

Physical activity is often quantified as physical activity energy expenditure (PAEE), which may be affected by individual ability to sustain physical activity intensity. We examined if there is a true difference in physical activity between older women and men when their physical capacity is considered.

**Methods:**

Out of the population-based sample of 1021 (75-85-year-old) adults (62 % women), 410 wore an accelerometer (100 Hz) and a single-channel ECG monitor (250 Hz) for at least 3 days in free-living. A treadmill walk calibration and previously validated equations were used to estimate PAEE from combined acceleration and heart rate sensing using branched equation modelling. Preferred walking speed in a six-minute walking test, fat free mass (FFM) using bioimpedance and isometric maximal leg extension strength scaled to FFM were measured. Independent t-test, Pearson correlation and hierarchical multiple regression were used for statistical analyses.

**Results:**

PAEE was 41.4 (14.2) kJ/kg/day for men, which was significantly higher (p < 0.001) than the PAEE 34.8 (10.9) for women. Walking speed (1.2 (0.2) vs. 1.1 (0.2) m/s), FFM (56.9 (6.5) vs. 42.1 (4.8) kg) and maximal strength (7.4 (1.8) vs. 6.7 (1.8) N/kg) were higher in men than in women (all p < 0.001). PAEE was significantly associated with walking speed (r = 0.44, p < 0.001), FFM (r = 0.26, p < 0.001), and maximal strength (r = 0.26, p < 0.001). In the regression analysis, 22 % of the variation in PAEE was explained by walking speed, FFM and maximal strength combined (F = 37.8, p < 0.001), whereas adding sex did not improve the model (F = 28.9, p < 0.001). The strongest predictor of PAEE was preferred walking speed (standardized beta=0.35, p < 0.001).

**Conclusions:**

The results indicate that the observed difference in physical activity volume between sexes was largely explained by physical characteristics, especially the faster preferred walking speed of men compared to women. Free-living physical activity is largely comprised of habitual walking especially in older people which makes walking speed a significant contributor of daily PAEE. Walking speed could be measured more widely in physical activity counselling and health care settings as a potential underlying factor of low physical activity in older people.

**Support/Funding Sources:**

European Research Council, Academy of Finland and Yrjö Jahnsson Foundation.

